# Trans-subclavian approach for radiofrequency ablation of premature ventricular contractions originating from subtricuspid annulus: a case report

**DOI:** 10.1186/1471-2261-13-7

**Published:** 2013-02-18

**Authors:** Teng Li, Xian-zhang Zhan, Ping-zhen Yang, Yu-mei Xue, Xian-hong Fang, Hong-tao Liao, Shu-lin Wu

**Affiliations:** 1 Cardiovascular Department, Guangdong Cardiovascular Institute, Guangdong General Hospital, No. 96, Dongchuan Road, Guangzhou 510080, China; 2 Department of Cardiology, Affiliated hospital of Guangdong Medical College, No. 57, Renming Road, Zhanjiang 524001, China

**Keywords:** Premature ventricular contractions, Tricuspid annulus, Radiofrequency catheter ablation

## Abstract

**Background:**

Catheter ablation has been established as a curative treatment strategy for ventricular arrhythmias. The standard procedure of most ventricular arrhythmias originating from the right ventricle is performed via the femoral vein. However, a femoral vein access may not achieve a successful ablation in some patients.

**Case presentation:**

We reported a case of a 29-year old patient with symptomatic premature ventricular contractions was referred for catheter ablation. Radiofrequency energy application at the earliest endocardial ventricular activation site via the right femoral vein could not eliminate the premature ventricular contractions. Epicardial mapping could not obtain an earlier ventricular activation when compared to the endocardial mapping, and at the earliest epicardial site could not provide an identical pace mapping. Finally, we redeployed the ablation catheter via the right subclavian vein by a long sheath. During mapping of the subvalvular area of the right ventricle, a site with a good pace mapping and early ventricular activation was found, and premature ventricular contractions were eliminated successfully.

**Conclusion:**

Ventricular arrhythmias originating from the subtricuspid annulus may be successfully abolished via a trans-subclavian approach and a long sheath. Although access via the right subclavian vein for mapping and ablation is an effective alternative, it is not a routine approach.

## Background

 The majority of idiopathic ventricular arrhythmias (VAs), including ventricular tachycardia and premature ventricular contractions (PVCs), have a right ventricular outflow tract or left ventricular outflow tract origin [1-4], but some originate from the aortic sinus cusp [5], coronary venous system [6], or mitral annulus [7]. A small number of cases of idiopathic VAs have been reported to originate from the tricuspid annulus [8]. Mapping and catheter ablation of the arrhythmias originating from the tricuspid annulus has been fully understood, and radiofrequency catheter ablation is an effective curative therapy for symptomatic PVCs originating from the vicinity of tricuspid annulus [9]. The standard catheter ablation procedure is performed via the femoral vein. In this case report, a trans-subclavian approach was used for the ablation of PVCs originating from the subtricuspid annulus of the right ventricle.

### Case presentation

A 29-year-old man was admitted with frequent premature ventricular contractions. Medical treatment with amiodarone, propafenone and beta-blocker was unsuccessful. He had no other current health problems or history of previous cardiovascular or other major diseases.

**Figure 1:**
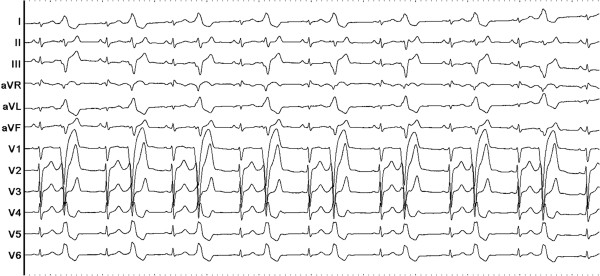
Twelve-lead ECG of the premature ventricular contractions (PVCs) which exhibited a left bundle branch block and left superior axis QRS morphology.

 A total number of 27,530 PVCs per day were observed on the 24-hour ambulatory Holter monitoring. At baseline, monomorphic PVCs were frequent and exhibited a left bundle branch block and left superior axis QRS morphology; upright R-waves in leads I, aVL; a rs pattern in lead II; a QS pattern in leads III, aVF, aVR and V1; and R-wave transition in the precordial lead V4 (Figure 1).

**Figure 2:**
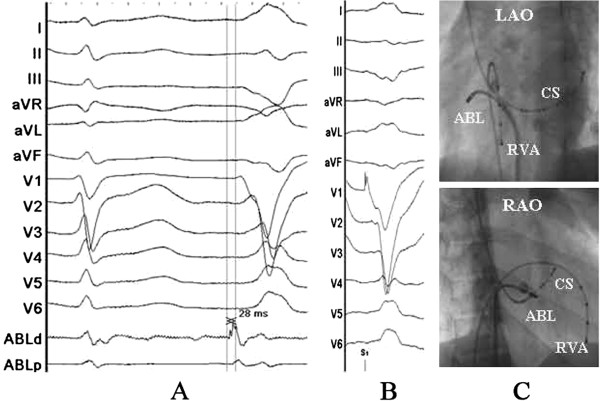
**Surface and intracardiac electrograms recordings, and fluoroscopic images obtained at the lateral subtricuspid annulus via the right femoral vein.** (**A**) The local ventricular activation time preceded the onset of the QRS complex was 24 ms. (**B**) Pace mapping at the same site. (**C**) Fluoroscopic images obtained in the left anterior oblique and right anterior oblique projection. ABL d (p) = the distal (proximal) electrode pair of ablation catheter; CS = coronary sinus; RVA = right ventricular apex.

**Figure 3:**
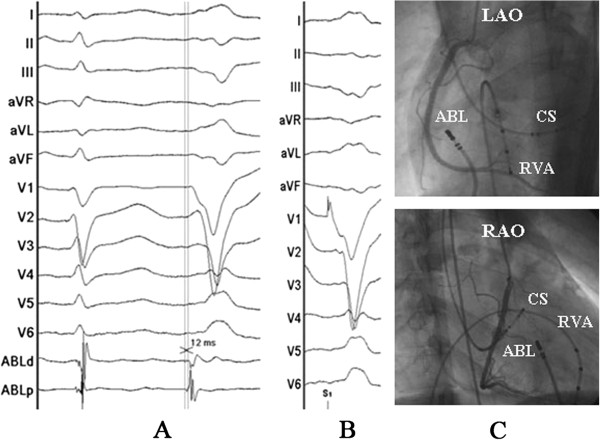
**Surface and intracardiac electrograms recordings, and fluoroscopic images obtained at the epicardial mapping site.** (**A**) The local ventricular activation time recorded at the epicardial site that preceded the onset of the QRS complex was 12 ms. (**B**) Pace mapping at the same site. (**C**) Fluoroscopic images obtained in the left anterior oblique and right anterior oblique projection. Abbreviations as indicated in Figure 2.

**Figure 4:**
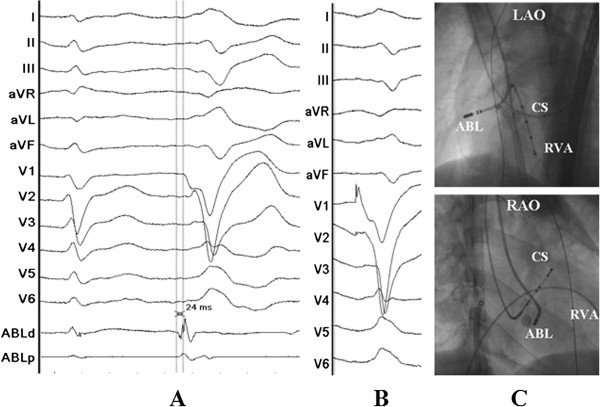
**Surface and intracardiac electrograms recordings, and fluoroscopic images obtained at the lateral subtricuspid annulus via the right subclavian vein.** (**A**) The local ventricular activation time preceded the onset of the QRS complex was 24 ms. (**B**) Pace mapping at the same site. (**C**) Fluoroscopic images obtained in the left anterior oblique and right anterior oblique projection showing the successful ablation site. Abbreviations as indicated in Figure 2.

**Figure 5:**
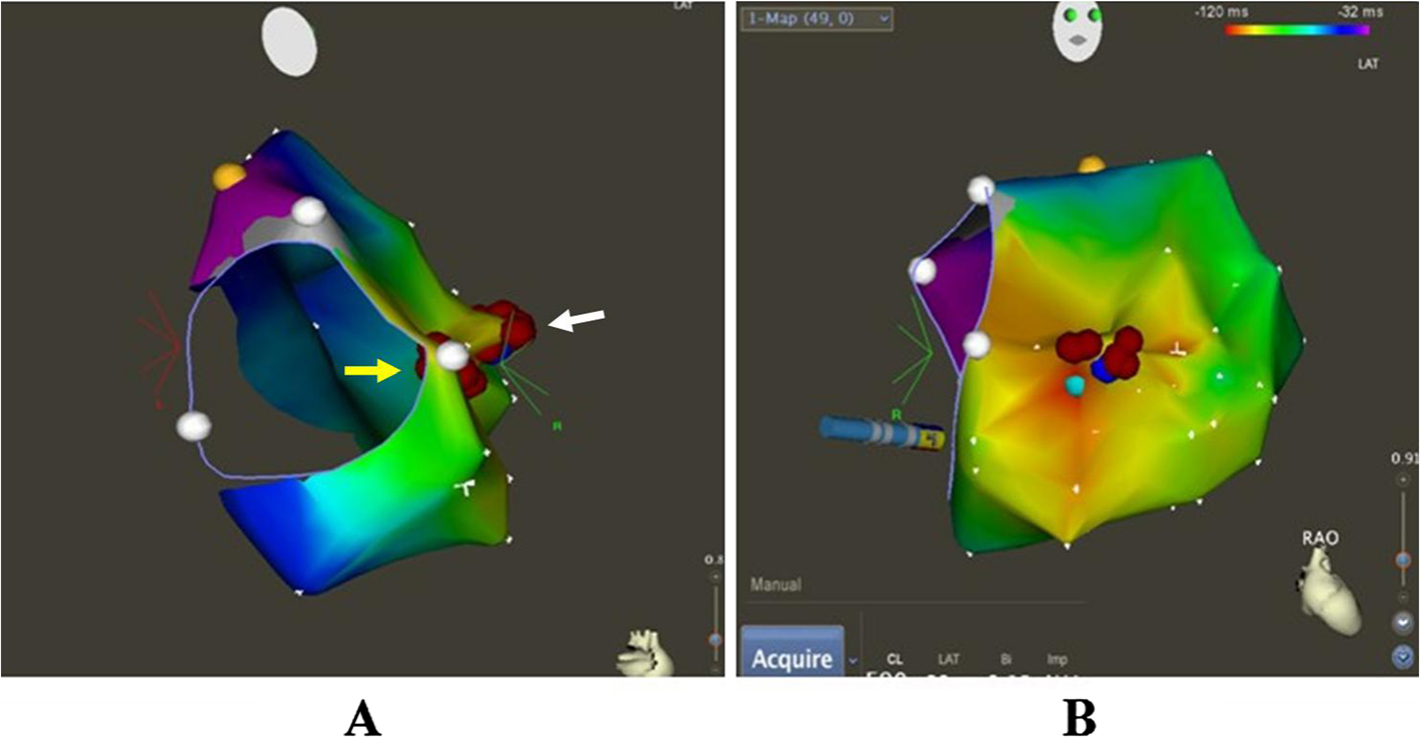
**CARTO system was used for activation mapping and identification of ablation site (A and B).** Successful ablation spots via the right subclavian vein (**A**, white arrow), unsuccessful ablation spots via the right femoral vein (**A**, yellow arrow). Red points = ablation spots; yellow point = his bundle; white points = tricuspid annulus.

**Figure 6:**
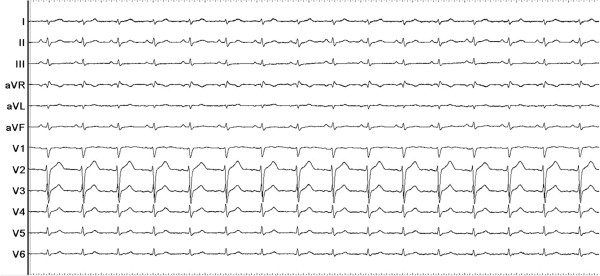
PVCs were not observed on the twelve-lead ECG after the procedure.

 Written informed consent was obtained, and the electrophysiologic study (EPS) was performed after all antiarrhythmic drugs were discontinued at least five half-lives before ablation. Under local anesthesia, a decapolar catheter was inserted into the coronary sinus (CS) from the left subclavian vein, and a quadripolar catheter was introduced from the right femoral vein and placed in the right ventricular apex (RVA). A long sheath (8-French, SR0) was used as needed for stabilizing via the right femoral vein, and a 3.5 mm, quadripolar cool saline irrigated catheter was used for mapping and ablation via the long sheath. The ablation procedure was guided by CARTO system. During activation mapping, the earliest activation was identified at the lateral subtricuspid annulus, preceding the QRS onset by 28 ms. The QRS morphology during pacing was similar to the spontaneous PVCs (Figure 2). Several radiofrequency applications (35 W, 43 C, 17 mL/min, 60 seconds) at the earliest activation site could abolish PVCs, but the PVCs had an immediate reoccurrence. Despite further applications around this site, the PVCs were still present. Despite the absence of ECG pattern evoking an epicardial origin, we decided to perform further ablation procedure using epicardial mapping through pericardial access. By percutaneous subxyphoid puncture, RF catheters could have been placed in the pericardial space for epicardial mapping. However, the earliest ventricular activation of epicardial mapping preceded the QRS onset was not earlier when compared to the endocardial mapping, and pace mapping could not provide an identical match with the PVCs morphology (Figure 3). Epicardial ablation was abandoned. Finally, we believed that it was because of difficulty in obtaining a stable contact of the ablation catheter. Ablation catheter was then reintroduced via the right subclavian vein; another long sheath (8-French, SR0) was used for stabilizing the ablation catheter to improve contact. During PVCs, the earliest activation was found close to the previous endocardial mapping site, preceding the onset of the QRS complex by 24 ms and pace mapping provided an identical match with the spontaneous PVCs (Figure 4A, B and C). A single radiofrequency application with a target temperature of 43°C and maximum power output of 35 W was delivered (Figure 5), which resulted in the successful abolition of the PVCs. Thereafter, no VAs could be induced despite a programmed electrical stimulation as well as an isoproterenol infusion (Figure 6). During more than 1 year of follow-up, the patient remained free of VAs without any antiarrhythmic drugs. No complications occurred during the ablation procedure or during a follow-up period. 

## Discussion

 The tricuspid annulus has been demonstrated to be one of the major sources of idiopathic VAs originating from the right ventricle [8]. However, to the best of our knowledge, there have been no reports describing frequent PVCs originating from the tricuspid annulus and in which catheter ablation abolished the PVCs via a trans-subclavian approach and a long sheath. For ablation of right-sided cardiac structures, ablation catheters are usually placed through a femoral vein. However, in certain clinical conditions, ablation may be very difficult or even impossible via femoral vein. Efficacy of the superior venous approach in the catheter ablation of atrioventricular reentrant tachycardia and atrial fibrillation had been reported [10,11]. Delivery of radiofrequency energy in target region resulted in the termination of PVCs via the femoral vein approach, but the PVCs had an immediate reoccurrence. Epicardial origin was suspected, but epicardial mapping could not obtain an earliest activation and pace mapping could not provide an identical match. Finally, we turned to a trans-subclavian approach. Because PVCs could be abolished and reoccurred soon during the first endocardial mapping and ablation, and this may due to an unstable contact of ablation catheter at the subtricuspid annulus via the right femoral vein. Then, the ablation catheter could easily reach the ablation site and have a perfect contact when we used a trans-subclavian approach, and the PVCs could be rapidly and effectively abolished without any reoccurrence via the trans-subclavian approach. Disadvantages of the superior approach such as greater radiation exposure to the ablationist and the challenge of manipulating a catheter and viewing intracardiac electrograms and fluoroscopy images from an unconventional angle have been reported [12]. We introduced a long pre-shaped sheath to avoid these disadvantages and achieved the same efficacy of the femoral vein approach.

## Conclusions

 The case report presented illustrates a case of access via the right subclavian approach and a long sheath for mapping and ablation of VAs originating from the subtricuspid annulus. Although access via the right subclavian vein for mapping and ablation is not a routine approach, when VAs originating from tricuspid annulus cannot be eliminated by RF application by the routine femoral vein approach, a trans-subclavian approach is an effective alternative, and a long pre-shaped sheath can be applied to avoid the disadvantages of the superior approach.

### Consent

 Written informed consent was obtained from the patient for publication of this case report and any accompanying images. A copy of the written consent is available for review by the Editor-in-Chief of this journal.

## Abbreviations

PVCs: Premature ventricular contractions; RF: Radiofrequency; VAs: Ventricular arrhythmias; EPS: Electrophysiologic study; CS: Coronary sinus; RVA: Right ventricular apex.

## Competing interests

The authors declare that they have no competing interests.

## Authors’ contributions

TL and XZZ have done the patient’s follow-up and drafted the manuscript. XZZ, PZY, HTL and XHF have done the ablation and provided the photographs of the ablation. YMX and SLW have helped on the manuscript drafting and revision. All authors read and approved the final manuscript.

## Pre-publication history

The pre-publication history for this paper can be accessed here:

http://www.biomedcentral.com/1471-2261/13/7/prepub
